# A comparison of the energy demands of quadrupedal movement training to walking

**DOI:** 10.3389/fspor.2022.992687

**Published:** 2022-10-13

**Authors:** Jeffrey D. Buxton, Sally A. Sherman, Micah T. Sterrett, Kristia D. Kannel, Morgan E. Blanchflower, Kelli T. Jancay, Anna K. Jenkins, Troy P. Donofrio, Philip J. Prins

**Affiliations:** ^1^Department of Exercise Science, Grove City College, Grove City, PA, United States; ^2^Department of Health and Human Development, University of Pittsburgh, Pittsburgh, PA, United States

**Keywords:** energy expenditure, physical activity, indirect calorimetry, transitional movements, Animal Flow

## Abstract

**Background:**

Quadrupedal movement training (QMT) is a novel alternative form of exercise recently shown to improve several fitness characteristics including flexibility, movement quality, and dynamic balance. However, the specific energy demands of this style of training remain unknown. Therefore, the purpose of this study was to compare the energy expenditure (EE) of a beginner-level quadrupedal movement training (QMT) class using Animal Flow (AF) to walking, and to compare EE between segments of the AF class and gender.

**Methods:**

Participants (15 male, 15 female) completed 60-min sessions of AF, treadmill walking at a self-selected intensity (SSIT) and treadmill walking at an intensity that matched the heart rate of the AF session (HRTM). Indirect calorimetry was used to estimate energy expenditure.

**Results:**

AF resulted in an EE of 6.7 ± 1.8 kcal/min, 5.4 ± 1.0 METs, and HR of 127.1 ± 16.1 bpm (63.4 ± 8.1% of the subjects' age-predicted maximum HR), while SSIT resulted in an EE of 5.1 ± 1.0 kcal/min, 4.3 ± 0.7 METs, HR of 99.8 ± 13.5 bpm (49.8 ± 6.7% age-predicted maximum HR), and HRTM resulted in and EE of 7.6 ± 2.2 kcal/min, 6.1 ± 1.0 METs, and HR of 124.9 ± 16.3 bpm (62.3 ± 8.2% age-predicted maximum HR). Overall, EE, METs, HR and respiratory data for AF was greater than SSIT (*p*'s < 0.001) and either comparable or slightly less than HRTM. The Flow segment showed the highest EE (8.7 ± 2.7 kcal/min), METs (7.0 ± 1.7) and HR (153.2 ± 15.7 bpm). Aside from HR, males demonstrated greater EE, METs, and respiratory values across all sessions and segments of AF than females.

**Conclusions:**

QMT using AF meets the ACSM's criteria for moderate-intensity physical activity and should be considered a viable alternative to help meet physical activity guidelines.

## Introduction

Physical activity (PA) in the form of aerobic (e.g., walking) and resistance training is recommended universally by nearly all public health organizations and scientific communities to combat rising rates of preventable diseases such as cardiovascular disease, diabetes, obesity, and others (1–3). Additionally, moderate to vigorous PA has been associated with a reduced risk of all-cause mortality regardless of how sedentary one may be (4). Unfortunately, adherence to PA guidelines globally remain low, with 28% of adults and nearly 80% of adolescents failing to meet the recommendations globally (5). Nationally, these numbers appear worse with only 23% of U.S. adults and 20% of adolescents meeting the PA guidelines (6). Accordingly, it is vital to continue to promote and identify the benefits (e.g., the metabolic demands) of various types of PA, including novel and alternative forms of PA, such as quadrupedal movement training (QMT), which may appeal to current and future generations and thus promote greater adherence.

Quadrupedal movement training (QMT) is a novel alternative method of training utilizing closed kinetic chain bodyweight exercises characterized by having the hands and feet in contact with the ground. This style of training has been used for several years in strength and conditioning and personal fitness within the dynamic warmups and conditioning segment of one's workout. Recently, several commercially available systems that utilize QMT have emerged, including Animal Flow™ (AF). The AF system consists of dynamic quadrupedal movements that are practiced, sequenced with other movements, and eventually choreographed into a flow (a series of AF movements linked together). Many of these movements resemble animal postures and movements including various quadrupedal crawling and rolling patterns and reflect the vast range of movements humans are capable of. Several of the movements used in AF and other QMT systems are offered as fitness options, but have also been used in physical rehabilitation of injuries and neurological diseases (7–11). The rise in popularity of QMT is evidenced by the recent formation of commercially available systems and, with specific regard to AF, its expansion to well over 8,000 instructors in over 50 countries since 2010.

Many QMT programs offered commercially are delivered in various formats including private instruction and group classes. AF group classes generally follow a progression through most or all the components of the AF system [(1) wrist mobilizations, (2) activations, (3) form specific stretches, (4) traveling forms, (5) switches and transitions, and (6) flow] with each component making up the “segments” of a group class (refer to SDC1 for more details on AF segments and movements). The physiological and metabolic benefits of group-based fitness activities is well documented (12–15). Unfortunately, there is a paucity of research available on AF or similar QMT systems. A recent study showed that 8 weeks of QMT using the AF system resulted in significantly greater improvements in functional movement screen scores, fundamental stability, and various active joint ranges of motion (16). Additionally, research has shown greater EMG activity of core muscles, proprioception and cognitive flexibility (the ability to effectively switch focus between 2 or more tasks) (17) following training using quadrupedal crawling exercises found in AF system (18, 19). This style of QMT appears to produce several fitness benefits, however, the metabolic demands of QMT and whether such style of training would meet current physical activity guidelines are unknown. This evidence and the rising popularity of QMT warrant further investigation.

Current evidence supports the use QMT to improve various fitness characteristics and cognitive function, however, a gap exists in the literature regarding the energy demands of this style of training. Understanding these demands will help to guide practitioners of its use as a viable alternative form of PA. Thus, the primary aim of this study was to quantify and compare the metabolic (energy) demands of a standardized 60-min beginner level AF session to traditional aerobic exercise (self-paced treadmill TM walking and heart-rate matched TM walking). Based on similar work that quantified the energy expenditure of yoga by Sherman et al. we hypothesized that QMT would result in greater energy expenditure (EE) than self-paced walking, but comparable or slightly less than matched intensity walking (20). A secondary aim of this study was to quantify and compare the metabolic demands of each segment of an AF session. Again, we hypothesized that there would be significant differences in EE between the various segments of the AF session. For each of these aims we also investigated the influence of sex, as research indicates differences in motor unit contractile properties, force generation muscle activation, fatigue and energy expenditure (21, 22). We hypothesized that EE across each session and each AF class segment would be greater for males than females.

## Materials and methods

### Experimental design

This study used a modified randomized crossover design to quantify the metabolic demands of a standardized AF class and to compare these to the demands of treadmill walking of the same duration at a self-selected intensity and a heart rate-matched (to the AF class) intensity. During the familiarization session, subjects were oriented to all testing instruments [heart rate (HR) monitors, exertion and affect scales, and the portable metabolic cart] and each of the AF movements that were used during the pre-recorded class. Following the familiarization session subjects were randomly assigned to one of the following experimental conditions that varied the order of exercise sessions (this sequence of exercises was chosen to ensure that the AF session always occurred prior to the HRTM session) (20):

Condition 1:

Session 1: AF session

Session 2: HR matched walking session

Session 3: Self-selected intensity walking session

Condition 2:

Session 1: AF session

Session 2: Self-selected intensity walking session

Session 3: HR matched walking session

Condition 3:

Session 1: Self-selected intensity walking session

Session 2: AF session

Session 3: HR matched walking session

During each of these trials, HR, METs, VO_2_, VCO_2_, RER, RR, Ve, Tv, and EE (kcal/min) were monitored continuously using a portable metabolic cart and heart rate monitor. Additionally, overall rating of perceived exertion (sRPE) and affect (sAffect) for each session were assessed at 5 min-post exercise. All familiarization and testing sessions were conducted in the Exercise Science Laboratory of Grove City College.

### Subjects

An *a priori* power analysis (G^*^Power 3.1.9.7) using moderate effect size and power set to 0.80 resulted in a suggested sample size of 28. A convenience sample of 30 subjects (male, *n* =15; female *n* = 15) naïve to QMT and AF volunteered for this study. Inclusion criteria include: (1) free of any physical limitations that would prohibit exercise; (2) currently participating in structured, moderate-intensity exercise at least 150 min per week, or 75 min of vigorous-intensity exercise per week, for at least 3 months; (3) between the ages of 18–35. Exclusion criteria include: (1) rostered collegiate athlete; (2) BMI ≥ 27 kg/m^2^; (3) currently smoke; (4) have known cardiovascular, metabolic, or pulmonary disease; (5) pregnant. Subjects were screened for health issues and physical limitations using a health history questionnaire. Before enrolling, all participants were informed of the risks and potential discomforts associated with testing and the intervention prior to providing their written informed consent to participate. The experimental protocol wad approved by the Institutional Review Board of Grove City College prior to implementation. Subjects were instructed to refrain from strenuous physical activity and caffeine consumption 24 h and food and drink 3 h prior to testing, and to refrain from the use of ergogenic aids throughout the study.

### Procedures

#### Familiarization sessions

Subjects completed a health history questionnaire and informed consent followed by anthropometric assessments including height (cm), mass (kg), fat free mass (kg), and fat mass (% and kg). Height was measured using a physician's scale (Detecto, Webb City, MO). Body mass and body composition (fat and lean mass) was measured using a Tanita bioelectrical impedance analyzer (BIA) (MC-980Uplus, Tanita Corporation of America, Arlington Heights, Illinois) following manufacturer procedures. The weight of subject's shorts and t-shirt was estimated at 0.5 kg and entered into the BIA. Subjects were instructed to remove their socks and shoes and then to stand on the BIA for ~30 s until the analysis was complete. Subjects were then fitted with a portable metabolic cart (K5, COSMED, Roma, Italy) and oriented to the treadmill (Trackmaster TMX425C treadmill, Newton, KS) and perceptual scales (OMNI Perceived Exertion Scale and Feeling Scale). Following this, subjects received instruction from the primary investigator on how to perform all the AF movements used in the pre-recorded AF session with proficiency being determined when subjects could perform each movement with minimal errors and cueing aside from a verbal call-out.

#### Experimental sessions

During each experimental session subjects were fitted with a heart rate monitor (Polar Electro, Kempele, Finland) at the level of their xiphoid process and the K5 portable metabolic cart (calibrated according to manufacturer guidelines prior to each experimental session). Subjects then sat quietly for 5 min to allow the K5 unit to complete start-up procedures prior to performing the AF or TM sessions. At the end of each session subjects were asked to rate their overall session rating of perceived exertion (sRPE) at 5-min post using the OMNI Walk/Run 0–10 Perceived Exertion Scale (23) and sAffect using the Feeling Scale (24). Each exercise session was 60 min long with a minimum of 48 h between sessions.

During the AF session (Table 1) subjects followed along to a pre-recorded AF workout video displayed on a large flat screen monitor, consisting of the following segments described previously (16): (1) wrist mobilizations (WM), (2) core and shoulder muscle activations (ACT), (3) form stretches (FS), (4) switches and transitional movements (STs), and (5) a choregraphed flow (FLOW) (refer to SDC1 for details on purpose of each segment and movements performed in each segment). Technique was monitored for each subject and if necessary, adjustments were made throughout the session to ensure proper technique and safety. Any necessary modifications (e.g., regressed version of an exercise or an extended rest period) were recorded by the investigators. All AF sessions were supervised by the principal investigator who is certified as a Level 1 Animal Flow™ instructor.

**Table 1:** Movements performed in the AF session.

**Segment**	**Movement**	**Reps/duration**
Wrist mobilizations	Wrist rolls	~30 s each direction
	Wrist wave	~30 s each direction
	Lateral wrist	5×
	Wrist rocks	5×
	Wrist circles	3–4× each direction
Activations	Beast 1	2 × ~10–30 s each
	Beast 2	2 × ~15 s each limb lift
	Beast 3	2 × ~10 s each limb lift
	Crab 1	1 × 60 s
	Crab 2	2 × ~15 s each limb lift
	Crab 3	2 × ~10 s each limb lift
Form stretches	Wave unload	2× (slow pace)
	Scorpion reach	2× each leg
	Crab reach	5–7 per arm
Switches and transitions	Reaching underswitch	3 × 30 s
	Full scorpion	3 × 30 s
	Jumping side kick through	3 × 30 s
	Front step through	3 × 30 s
Flow	Deep ape, reaching underswitch, full scorpion, jumping side kick through, jump to crab reach, underswitch to loaded beast, front step through, pop to loaded beast, pop to deep ape	4 × to right side only, then
		3 × to left side only, then
		3 × full flow (continuous from right side to left side)

Procedures for both the HR-matched (HRTM) and Self-selected intensity walking sessions (SSIT) were the same as those described in Sherman et al. (20). For the HRTM sessions, target HRs were determined by averaging the minute-by-minute HRs obtained during each segment of the AF session. These target HRs were obtained and maintained during the HRTM session in accordance with the timestamps and durations of each segment of AF session. For example, the average HR obtained during the WM segment of the AF session, which lasted roughly 5 min, was obtained and maintained for the first 5 min of the HRTM (and so on) using the following procedures. The session began with the treadmill set at a speed of 0.9 m/s (2.0 mph) and 0% incline. Every 30 s, the speed of the treadmill was increased by 0.09 m/s (0.2 mph) until the subject achieved the target heart rate (±5 bpm). After the initial 5 min, adjustments were made to the speed of the treadmill as needed throughout each time segment to maintain the heart rate within the calculated target range. Once a speed of 1.8 m/s (4.0 mph) was reached, incline was adjusted in 0.5% increments in order to maintain the desired heart rate range. This protocol was repeated for each corresponding AF session segment time to adjust for the changes in HR and maintain matching to the AF session.

For the SSIT walking session, subjects began walking on the treadmill at a speed of 0.4 m/s (1.0 mph) and 0% grade. During the initial 5 min speed was either maintained or increased or decreased by 0.2 m/s (0.5 mph) every 30 s to elicit the subject's perceived comfortable brisk-walking pace. The speed achieved after the initial 5-min period was maintained throughout the trial unless the subject requested to change it. Additionally, subjects were asked if they desired a change in walking speed every 5 min. During both treadmill walking sessions, the speed and incline information on the treadmill display were covered to eliminate any potential influence that this would have on the subject.

### Statistical analysis

Statistical analyses were performed using SPSS version 26.0 (SPSS Inc., Chicago, IL). Statistical significance was set *a priori* at *p* < 0.05. Descriptive statistics were calculated for all physiological and metabolic variables. Data was tested for normality using the Shapiro-Wilk test. Independent samples *t*-tests were used to evaluate differences in demographic characteristics between genders. A 2 (gender; male and female) × 3 (session; SSIT, HRTM, and AF) repeat measures ANOVA was performed to compare differences in mean HR, sRPE, sAffect and metabolic data between AF and TM sessions. A 2 (gender) × 5 [segment; WM (wrist mobility), ACT (activations), FS (form stretches), STs (switches and transitions), and FLOW (flow)] repeat measures ANOVA was performed to compare differences between segments of the AF session. Bonferroni *post-hoc* assessments were used to examine significant main and interaction effects. Independent *t*-tests were used for sub-analysis of any gender differences for each session and segment of the AF session. The assumption of sphericity was confirmed using Mauchly's test. Greenhouse-Geisser epsilon corrections were used when the assumption of sphericity was violated. Effect sizes were calculated using partial eta squared ($\eta_{\text{p}}^{2}$) (small = 0.01, medium = 0.06, and large = 0.14).

## Results

### Demographic information

Table 2 displays the descriptive characteristics of the subjects and differences in characteristics between genders. There was no difference between males and females for fat mass (*p* = 0.569). Males were overall taller, heavier, and had a greater BMI and fat free mass than females (*p*'s ≤ 0.003). Females displayed significantly greater body fat percentage (*p* = 0.001). All participants completed the AF session without the need for any modifications.

**Table 2:** Physical characteristics (means ± SD) of subjects (*n* = 30).

**Characteristic**	**Total (*n* = 30)**	**Males (*n* = 15)**	**Females (*n* = 15)**	***p*-value**
Age (yrs)	19.5 ± 1.4	20.1 ± 1.5^*^	19.1 ± 1.2	0.042
Height (cm)	174.0 ± 9.3	178.3 ± 8.3^*^	168.8 ± 7.9	0.003
Mass (kg)	73.2 ± 13.0	82.0 ± 11.1^*^	62.7 ± 10.1	<0.001
BMI (kg/m^2^)	24.0 ± 2.7	25.7 ± 2.6^*^	21.9 ± 2.1	<0.001
Body fat (%)	18.6 ± 4.8	15.7 ± 4.6	21.8 ± 3.8^*^	0.001
Fat mass (kg)	13.4 ± 3.6	13.0 ± 4.2	13.9 ± 4.4	0.549
Fat free mass (kg)	59.6 ± 12.5	69.0 ± 9.5^*^	48.6 ± 6.5	<0.001

* p < 0.05, significantly greater.

### Analysis of differences between sessions and gender

#### Energy cost

**Figure 1:**
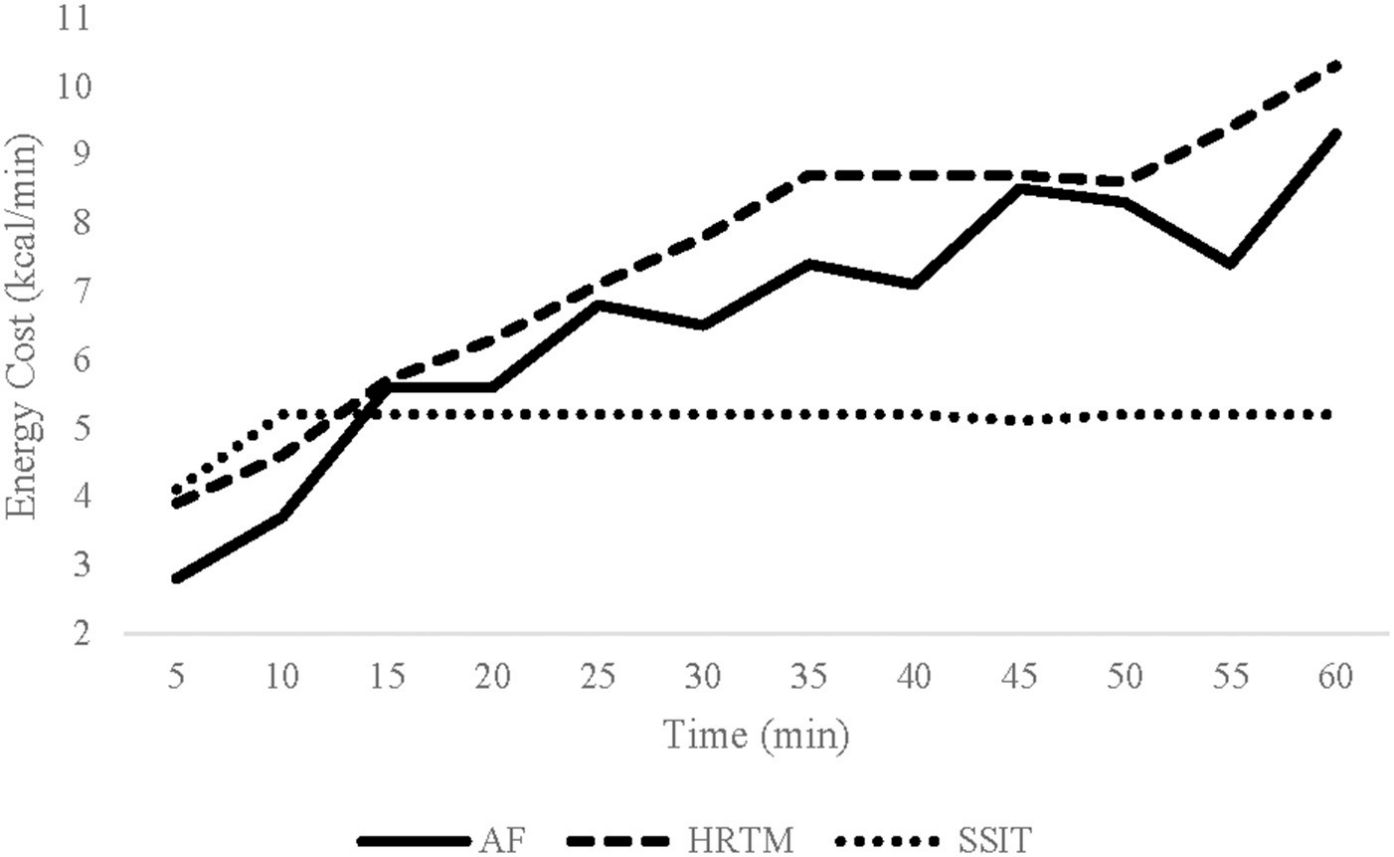
Average Kcal/min across each condition (AF, HRTM, SSIT). AF, Animal Flow; HRTM, heart rate matched treadmill walking; SSIT, self-selected intensity treadmill walking.

There were significant interactions for energy cost (kcal/h, kcal/min, and kcal/session; *p*'s ≤ 0.010). AF and HRTM sessions resulted in significantly higher energy cost than SSIT (*p*'s < 0.001) and HRTM sessions resulted in higher energy cost than AF (*p*'s ≤ 0.033) (Table 3; Figure 1). Sub-analysis using independent *t*-tests showed that energy cost was significantly higher for males than females for all sessions (*p*'s < 0.001; Table 3).

**Table 3:** Comparison of HR, METs, energy expenditure, and perceptual data across sessions (AF, SSIT, HRTM) (*n* = 30).

**Variable**	**SSIT**	**HRTM**	**AF**	***P*-Value**
HR (b^.^min^−1^)	99.8 ± 13.5	124.9 ± 16.3^*^	127.1 ± 16.1^*^	Interaction; *P* = 0.853, η^p2^ = 0.004
Male	94.3 ± 10.2	119.3 ± 15.9	121.1 ± 14.8	Session; *P* < 001, η^p2^ = 0.852
Female	105.5 ± 14.1^°^	130.0 ±16.1	133.2 ± 16.4^°^	Gender; *P* = 0.038, η^p2^ = 0.155
METs	4.3 ± 0.7	6.1 ± 1.0^*^^‡^	5.4 ± 1.0^*^	Interaction; *P* = 0.081, η^p2^ = 0.089
Male	4.2 ± 0.6	6.5 ± 1.0	5.8 ± 1.0^•^	Session; *P* < 001, η^p2^ = 0.614
Female	4.3 ± 0.7	5.7 ± 0.9	5.0 ± 0.9	Gender; *P* < 0.048, η^p2^ = 0.137
Kcal/h	308.2 ± 62.3	453.5 ± 134.6^*^^‡^	403.0 ± 106.7^*^	Interaction; *P* = 0.008, η^p2^ = 0.164
Male	351.2 ± 43.6^•^	546.0 ± 105.5^•^	486.6 ± 65.8^•^	Session; *P* < 001, η^p2^ = 0.613
Female	269.4 ± 52.6	367.2 ± 96.7	323.1 ± 75.2	Gender; *P* < 0.001, η^p2^ = 0.614
Kcal/min	5.1 ± 1.0	7.6 ± 2.2^*^^‡^	6.7 ± 1.8^*^	Interaction; *P* = 0.008, η^p2^ = 0.164
Male	5.9 ± 0.7^•^	9.1 ± 1.8^•^	8.1 ± 1.1^•^	Session; *P* < 001, η^p2^ = 0.613
Female	4.5 ± 0.9	6.1 ± 1.6	5.4 ± 1.8	Gender; *P* < 0.001, η^p2^ = 0.614
Kcal/session	309.8 ± 63.5	457.8 ± 135.3^*^^‡^	411.8 ± 109.7^*^	Interaction; *P* = 0.010, η^p2^ = 0.157
Male	353.9 ± 44.6^•^	549.6 ± 107.9^•^	496.9 ± 69.0^•^	Session; *P* < 001, η^p2^ = 0.616
Female	270.8 ± 53.3	372.0 ± 97.4	330.7 ± 78.4	Gender; *P* < 0.001, η^p2^ = 0.603
sRPE	2.0 ± 1.2	5.7 ± 1.4^*^	6.5 ± 1.8^*^^†^	Interaction; *P* = 0.878, η^p2^ = 0.005
Male	1.8 ± 0.9	5.6 ± 1.7	6.3 ± 1.9	Session; *P* < 001, η^p2^ = 0.769
Female	2.3 ± 1.3	5.7 ± 1.2	6.7 ± 1.7	Gender; *P* < 0.379, η^p2^ = 0.028
sAffect	4.1 ± 1.3^†^^‡^	2.6 ± 1.9	2.9 ± 2.0	Interaction; *P* = 0.857, η^p2^ = 0.005
Male	4.2 ± 0.9	2.7 ± 2.2	2.8 ± 1.8	Session; *P* < 001, η^p2^ = 0.246
Female	3.9 ± 1.7	2.6 ± 1.7	2.9 ± 2.3	Gender; *P* < 0.898, η^p2^ = 0.001

Data are Mean ± SD.

SPTM, self-paced treadmill walking; HRTM, heart-rate matched treadmill walking; AF, Animal Flow; HR, heart rate; sRPE, session rating of percieved exertion; sAffect, session affect; Effect sizes: 0.01 = small; 0.06 = moderate; ≥ 0.14 = large.

* Significantly greater than SSIT.

† Significantly greater than HRTM.

‡ Significantly greater than AF.

• Significantly greater than females.

° Significantly greater than males.

#### Heart rate and METs

**Figure 2:**
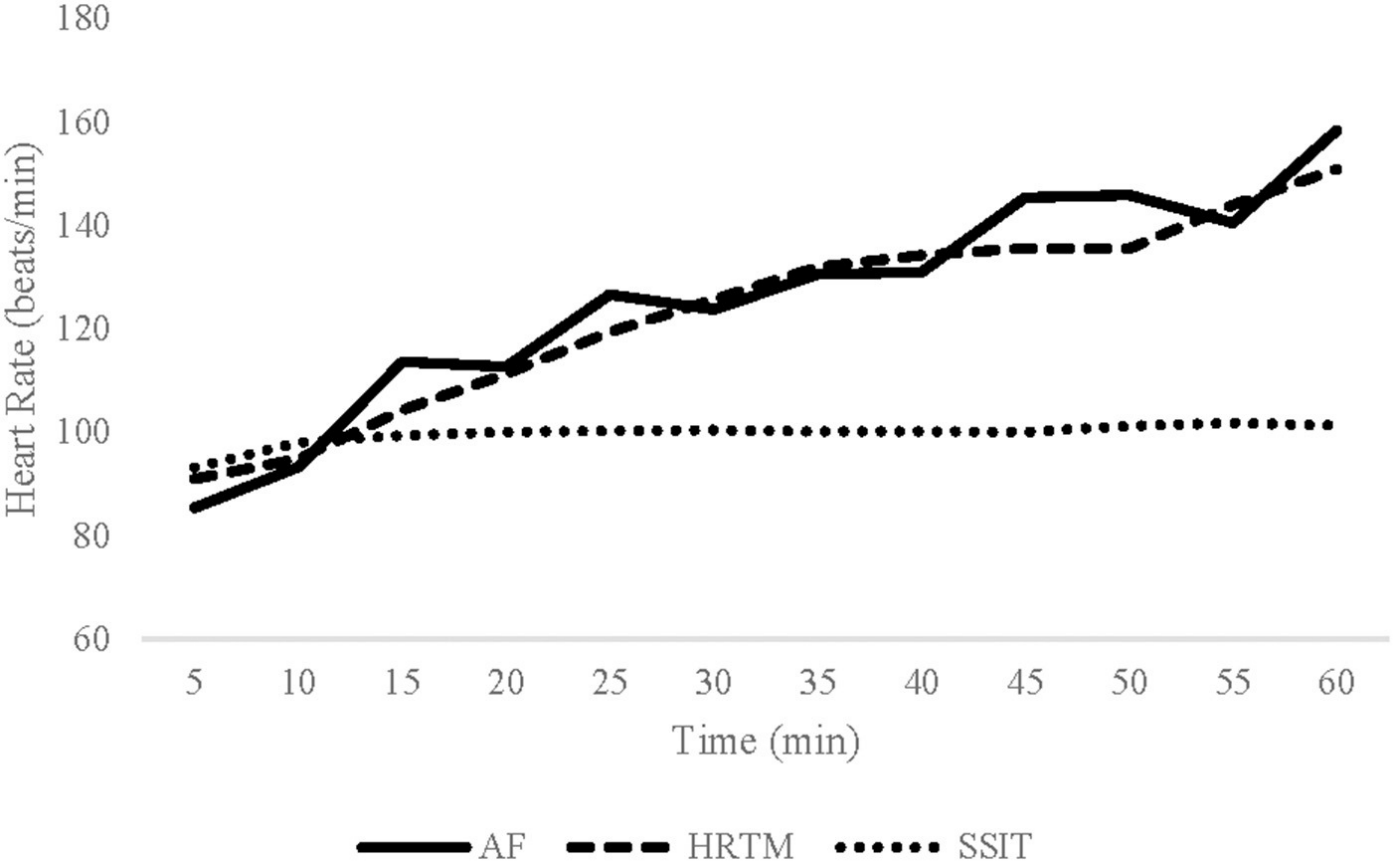
Average heart rate (bpm) responses across each condition (AF, HRTM, SSIT). AF, Animal Flow; HRTM, heart rate matched treadmill walking; SSIT, self-selected intensity treadmill walking.

There was no significant interaction for HR. There was a main effect for session (*p* < 0.001) with *post-hoc* analysis showing that HRTM and AF sessions resulted in significantly higher average session HR than SSIT (*p*'s < 0.001) and there was no difference in HR between HRTM and AF (Table 3; Figure 2). Additionally, there was a significant main effect of gender (*p* = 0.038) with sub-analysis revealing significantly higher heart rates for females during the SSIT and AF sessions (*p*'s < 0.033).

There was no significant interaction for METs however, there was a significant main effect of gender (*p* = 0.48) and session (*p* < 0.001). *Post-hoc* analysis showed that METs were significantly higher in the HRTM and AF sessions than SSIT (*p*'s < 0.001) and HRTM resulted in significantly higher METs than AF (*p* = 0.014). Additionally, METs was significantly higher for males than females in the AF session (Table 3).

#### Perceptual data

There were no significant interactions or main effects of gender for sRPE and sAffect. There was a significant main effect of session (*p*'s < 0.001). *Post-hoc* testing showed AF had higher sRPE than both HRTM (*p* = 0.042) and SPTM (*p* < 0.001) and HRTM was higher than SPTM (*p* < 0.001). SSIT resulted in higher sAffect than HRTM (*p* < 0.001) and AF (*p* = 0.015), with no differences between AF and HRTM (Table 3).

### Analysis of Animal Flow segments by gender

#### Energy cost

**Figure 3:**
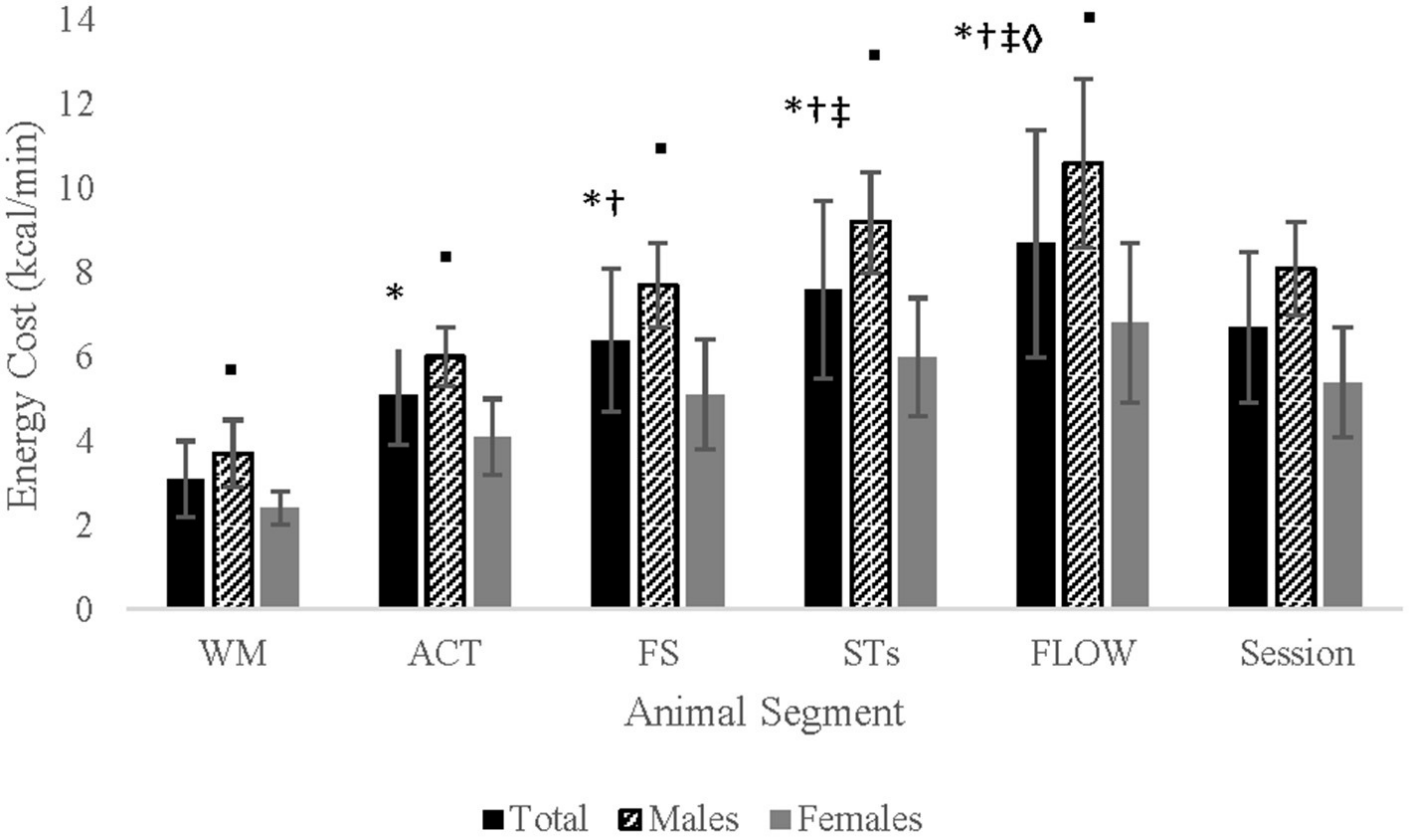
Kcal/min between genders for each segment of the Animal Flow session. WM, wrist mobilizations; ACT, activations; FS, form stretches; STs, switches and transitions; FLOW, choreographed flow; *significantly greater than WM; ^†^significantly greater than ACT; ^‡^significantly greater than FS; ^♢^significantly greater than STs; °males significantly greater than females.

There was a significant interaction between gender and segment for energy cost (kcal/segment, kcal/hour, and kcal/min) (*p*'s < 0.001). Energy cost (kcal/h and kcal/min) significantly increased from one segment to the next with males demonstrating a significantly greater energy cost during each segment than females. Total kcals/segment significantly increased from segment 1–4 (*p*'s < 0.001). Segment (5) resulted in significantly greater kcals than segment 1–3 but < 4 (*p*'s < 0.001; Table 4; Figure 3).

**Table 4:** Comparison of HR, METs, and energy expenditure across segments of AF (*n* = 30).

**Variable**	**WM**	**ACT**	**FS**	**STs**	**FLOW**	***P*-value**
HR (b^.^min^−1^)	86.2 ± 15.4	108.0 ± 16.4^*^	120.6 ± 18.9^*^^†^	134.7 ± 17.7^*^^†^^‡^	± 15.7^*^^†^^‡^^♢^	Interaction; *P* = 0.107, 0.107, η^p2^ = 0.074
Males	82.1 ± 12.7	99.0 ± 10.6	112.9 ± 14.8	128.4 ± 16.6	147.8 ± 15.8	Session; *P* < 001, η^p2^ = 0.937
Females	89.9 ± 12.2	116.3 ± 16.6^°^	127.9 ± 19.8	140.5 ± 17.3	158.3 ± 14.2	Gender; *P* = 0.029, η^p2^ = 0.165
METS	2.5 ± 0.6	4.0 ± 0.6^*^	5.1 ± 1.0^*^^†^	6.1 ± 1.2^*^^†^^‡^	7.0 ± 1.7^*^^†^^‡^^♢^	Interaction; *P* = 0.097, η^p2^ = 0.090
Males	2.7 ± 0.7^■^	4.3 ± 0.5^■^	5.5 ± 0.9^■^	6.7 ± 1.0^■^	7.7 ± 1.5^■^	Session; *P* < 001, η^p2^ = 0.898
Females	2.3 ± 0.3	3.8 ± 0.6	4.8 ± 0.9	5.6 ± 1.1	6.4 ± 1.6	Gender; *P* = 0.012, η^p2^ = 0.213
Kcal/hour	181.9 ± 55.0	301.0 ± 73.2^*^	380.0 ± 103.6^*^^†^	453.5 ±125.1^*^^†^^‡^	519.2 ± 163.2^*^^†^^‡^^♢^	Interaction; *P* = 0.001, η^p2^ = 0.293
Males	223.4 ± 46.9^■^	357.3 ± 40.2^■^	458.5 ± 64.3^■^	551.9 ± 74.0^■^	636.7 ± 122.5^■^	Session; *P* < 001, η^p2^ = 0.892
Females	143.2 ± 26.2	248.5 ± 55.6	306.6 ± 75.4	361.6 ± 86.4	409.5 ± 112.4	Gender; *P* < 0.001, η^p2^ = 0.608
Kcal/min	3.0 ± 0.9	5.0 ± 1.2^*^	6.3 ± 1.7^*^^†^	± 2.1^*^^†^^‡^	8.7 ± 2.7^*^^†^^‡^^♢^	Interaction; *P* = 0.001, η^p2^ = 0.290
Males	3.7 ± 0.8^■^	6.0 ± 0.7^■^	7.6 ± 1.1^■^	9.2 ± 1.2^■^	10.6 ± 2.0^■^	Session; *P* < 001, η^p2^ = 0.891
Females	2.4 ± 0.4	4.1 ± 0.9	5.1 ± 1.3	6.0 ± 1.4	6.8 ± 1.9	Gender; *P* < 0.001, η^p2^ = 0.609
Kcal/segment	20.6 ± 7.7	45.1 ± 12.7^*^	61.0 ± 17.7^*^^†^	185.4 ± 50.6^*^^†^^‡^^•^	97.4 ± 29.7^*^^†^^‡^	Interaction; *P* < 0.001, η^p2^ = 0.475
Males	25.6 ± 8.1	53.9 ± 6.8	74.6 ± 12.1	223.9 ± 32.0	118.7 ± 20.6	Session; *P* < 001, η^p2^ = 0.958
Females	15.9 ± 3.0	36.9 ± 11.4	48.3 ± 11.8	149.4 ± 35.8	77.5 ± 22.1	Gender; *P* < 0.001, η^p2^ = 0.581

Data are Mean ± SD. AF, Animal Flow; WM, wrist mobilizations; ACT, activations; FS, form Data are Mean ± SD.

AF, Animal Flow; WM, wrist mobilizations; ACT, activations; FS, form stretches; STs, switches and transitions; FLOW, choreographed flow; HR, heart rate; METs, metabolic equivalents; Effect sizes: 0.01 = small; 0.06 = moderate; ≥ 0.14 = large.

* Significantly greater than WM.

† Significantly greater than ACT.

‡ Significantly greater than FS.

♢ Significantly greater than STs.

• Significantly greater than FLOW.

■ Significantly greater than females.

° Significantly greater than males.

#### Heart rate and METs

**Figure 4:**
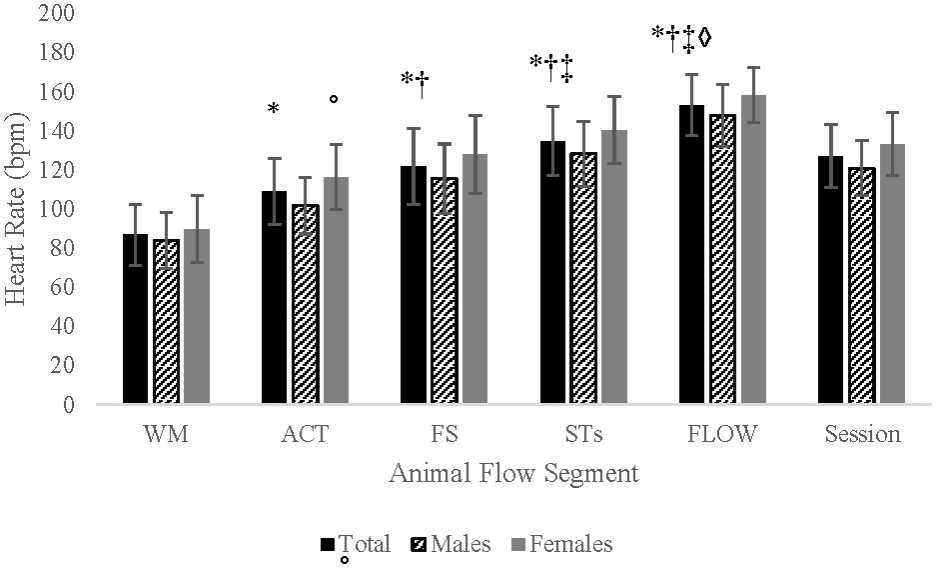
Heart rate comparisons between Animal Flow segments. Data are Mean ± SD. WM, wrist mobilizations; ACT, activations; FS, form stretches; STs, switches and transitions; FLOW, choreographed flow; *significantly greater than WM; ^†^significantly greater than ACT; ^‡^significantly greater than FS; ^♢^significantly greater than STs; °females significantly greater than males.

There was no significant interaction between gender and segment for HR. There was a significant main effect of segment (*p* < 0.001) with *post-hoc* analysis showing that HR increased from one segment to the next (*p*'s < 0.001). There was a significant main effect of gender (*p* = 0.029) with sub-analysis showing a significantly higher HR for females than males for segment 2 (*p* = 0.015; Table 4; Figure 4). Similarly, there was no sig interaction for METs but a significant main effect for session (*p* < 0.001) and gender (*p* = 0.012). *Post-hoc* analysis showed that METs significantly increased from one segment to the next (*p*'s < 0.001) and METs was greater for males than females for each segment (*p*'s ≤ 0.035; Table 4).

## Discussion

The purpose of this study was to compare the energy cost and metabolic demands of a beginner level QMT program (using Animal Flow™) to two types of treadmill walking and to explore the influence of gender across all outcomes. QMT using AF resulted in an average energy expenditure of 6.7 ± 1.8 kcal/min (411.8 ± 106.7 kcal/session) and 5.4 ± 1.0 METs, which meets the criteria to be considered moderate intensity physical activity, typically defined as 3 to < 6.0 METs (25). QMT resulted in an average HR of 127.1 ± 16.1 bpm which was 63.4 ± 8.1% of the subjects' age-predicted maximum HR (APMHR: 220—age), again, reflecting moderate intensity exercise (26). The energy costs, MET values, HR, and respiratory data (VO_2_, VCO_2_, Ve, RR, Tv, and RER: refer to SDC2) for QMT was greater than self-selected intensity walking and either comparable or slightly less than matched-heart rate walking. Greater energy costs and overall metabolic demands were noticed in males across all sessions, most likely due to their greater overall body mass and fat-free mass (27). Interestingly, females demonstrated significantly higher average session heart rates (~10 bpm) than males for the SSIT and QMT session. This may be explained by recent work suggesting that females demonstrate different motor unit recruitment strategies than males. Specifically, that females have higher motor unit discharge rates at low forces (<40% MVIC) compared to males who appear to have higher motor unit discharge rates at higher forces (28). At present it is unknown what % of MVIC is elicited during QMT, but most likely forces are submaximal in general. As such, this may have resulted in increased neural drive and demand in females resulting in elevated HR during QMT. It is unclear why HR was different between genders during the SSIT.

Being the first study to investigate the energy costs of QMT there are no other studies to use as a direct comparison. However, the metabolic demands of several other novel and/or alternative forms of exercise have been investigated including yoga (20, 29, 30) and pole dancing (31). Both practices share similarities in class design to AF as they generally begin with a gradual warmup, followed by a skills segment and a routine-segment. Overall, the energy expenditures in this study are similar to those found in yoga and pole dancing. Sherman et al. found that 60 min of vinyasa yoga resulted in 4.8 kcal/min and 3.7 METs (5.2 kcal/min and 4.1 METs when the restorative component of the session was left out of analysis). Hagins et al. found slightly lower energy costs (3.2 kcal/min) (29) while Tsopanidou et al. found higher energy costs (7.1 kcal/min) potentially due to the inclusion of a “high intensity vinyasa” segment (30). Similarly, in their recent study, Nicholas et al. found that a 60-min Pole dancing class resulted in an average energy expenditure of 4.7 kcal/min and 4.6 METs (31).

The finding that QMT using AF resulted in moderate intensity physical activity is important for public health as this intensity of exercise is recommended as a means for reducing the risk of chronic conditions such as cardiovascular disease, obesity, diabetes, and others (4, 32). Although the benefits of QMT are not well known, Buxton et al. demonstrated that QMT can improve active joint flexibility, movement quality, and dynamic balance (16). Additionally, Matthews et al. showed QMT enhanced cognitive flexibility and proprioception (18). Given these findings, QMT using the AF system appears to be a viable alternative form of exercise capable of potentially providing numerous health benefits.

A secondary purpose of this study was to compare the energy demands of each segment of the QMT program and explore any gender differences therein. The energy cost (kcal/min) significantly increased from the first to the last segment with the Flow segment resulting in the highest energy expenditure (8.7 ± 2.7 kcal/min). Similarly, METs and heart rate significantly increased from the first to last segment with the Flow segment demonstrating the highest value for each (7.0 ± 1.7 and 153.2 ± 15.7 bpm, respectively). This increase in EE, METs, and HR from the first segment of the QMT class (wrist mobilizations) to the last (choreographed flow) is most likely due to a combination of accumulated muscular fatigue and the increased intensity associated with each segment. In general, males demonstrated significantly greater energy costs, METs and respiratory values (VO_2_, VCO_2_, Ve, Tv, RR, and RER) than females across each segment, again most likely due to the reasons mentioned earlier. Heart rates were higher overall (regardless of segment) for females and were significantly higher than males during segment 2 (Activations). Again, this may be due to differences in motor unit recruitment strategies discussed previously.

The wrist mobilization segment resulted in HR values of 43.4 ± 7.9% of subjects' APMHR and 2.5 ± 0.6 METs representing light intensity (~11% of AF class). The activations segment resulted in 54.44 ± 8.5% of subjects' APMHR and 4.0 ± 0.6 METs, indicating moderate intensity (~15% of class). The form specific stretches segment resulted in 60.8 ± 9.8% of APMHR and 5.1 ± 1.0 METS, again indicating moderate intensity (~15% of class). The switches and transitions segment resulted in 67.2 ± 8.9% of APMHR and 6.1 ± 1.2 METs, indicating vigorous intensity (~40% of class). Lastly the flow segment resulted in 76.4 ± 7.9% of APMHR and 7.0 ± 1.7 METs, again indicating vigorous intensity (~19% of class). Overall, about 40% of the QMT class was light to moderate intensity while roughly 60% of the class was considered vigorous intensity. It is important to note that a typical class format like the one used for this study is intermittent in nature rather than continuous. Additionally, this data shows that target intensities may vary depending on how a QMT class is configured with respect to these segments. Understanding the general intensity category for these segments can help guide individuals programing QMT using the AF system. Depending on individual/group goals and fitness levels, the exercise professional can manage how much time is spent in each segment to meet specific target training intensities. Additionally, this information can help inform future studies using different populations (e.g., older adults, overweight/obese, etc.) and fitness abilities.

## Limitations

There are several limitations of the present study worth noting which may impact the interpretation or usefulness of its findings. Subjects were required to perform each session while wearing a portable metabolic cart and face mask. It is unknown if wearing this equipment may have influenced energy expenditure. Additionally, subjects completed all exercise sessions in a controlled lab environment and in a non-group/class setting. It is unclear if the results of this study would be similar in a non-clinical, typical group class setting. To our knowledge AF is one of the leading systems of QMT, however several other systems exist. Whether the energy demands from this study would be similar to other forms of QMT remains unclear. Finally, the subjects in this study were healthy active college-aged individuals naïve to QMT. Therefore, our results may not be generalizable to other populations or more skilled practitioners of QMT. Each of these limitations present opportunities for future research.

## Conclusions

This was the first study to investigate the energy demands of a typical 60-min beginner level QMT class using the Animal Flow system. QMT resulted in an average energy expenditure of nearly 7 kcal/min and a MET level of 5.4, meeting the criteria to be considered moderate intensity exercise (25). This intensity is associated with several health benefits including the potential to reduce the risk of certain chronic conditions (1–4) and thus expanding upon the previously identified benefits of QMT including increased flexibility, movement quality, dynamic balance and muscular endurance (16). Finally, this study highlights differences in energy demands and intensity between the various segments of Animal Flow suggesting that the configuration of these segments in a QMT class/workout will largely influence overall energy demand and intensity. Given these findings, QMT using AF presents a viable alternative form of physical activity potentially capable of eliciting several important health benefits and provides justification for expanding recommendations to include this style of QMT to meet physical activity guidelines.

## Data availability statement

The raw data supporting the conclusions of this article will be made available by the authors, without undue reservation.

## Ethics statement

The studies involving human participants were reviewed and approved by Grove City College Institutional Review Board. The patients/participants provided their written informed consent to participate in this study.

## Author contributions

JB and SS gave substantial contributions to the conception and the design of the manuscript. JB, SS, MS, KK, MB, KJ, AJ, TD, and PP have given substantial contribution to acquisition and analysis and interpretation of the data. All authors have participated to drafting the manuscript and JB revised it critically. All authors read and approved the final version of the manuscript.

## Funding

This research was funded by a Grant from Grove City College Swezey Fund.

## Conflict of interest

The authors declare that the research was conducted in the absence of any commercial or financial relationships that could be construed as a potential conflict of interest.

## Publisher's note

All claims expressed in this article are solely those of the authors and do not necessarily represent those of their affiliated organizations, or those of the publisher, the editors and the reviewers. Any product that may be evaluated in this article, or claim that may be made by its manufacturer, is not guaranteed or endorsed by the publisher.
