# Supplementary material for: A comparison of the energy demands of quadrupedal movement training to walking
Source: Front Sports Act Living. 2022 Oct 13;4:992687. doi: 10.3389/fspor.2022.992687 (PMC9606455; doi:10.3389/fspor.2022.992687)
Supplement: Supplementary file 1 [file Data_Sheet_1.docx]

**Supplemental Digital Content 1: Description of Animal Flow Components/Segments and Movements Used in the Beginner Level Group Class**

**Wrist Mobilizations**: Wrist mobilizations are simple movements including static and active stretches to prepare the hands and wrists for the demands of the AF session.

**Activations:** Activation exercises are static holds (much like a plank) using two of the base positions (Beast and Crab) with attention given to proper alignment throughout the holds. These exercises serve to challenge the stabilization systems of the trunk, shoulder girdle and hip.

*Beast Activations*

1. Static beast – Begin on all fours with hands directly under shoulders and knees under or slightly in front of hips. Spine, head, neck and shoulder blades should be neutrally aligned. Raise knees slightly off the ground and hold position.


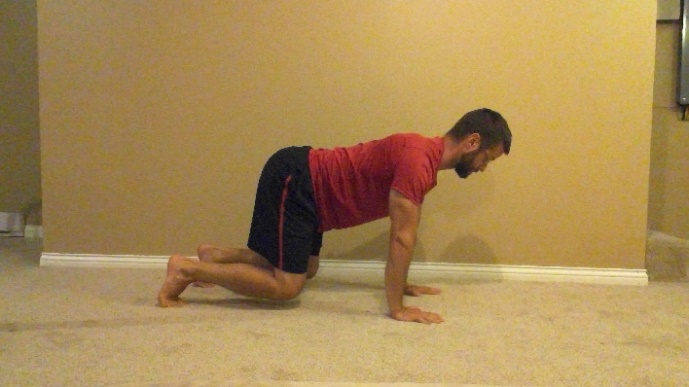


2. Static with single limb lift – Set Static Beast then lift a single limb (hand or foot) off the ground only high enough to slide a piece of paper under the hand or foot without letting any other body movements occur.


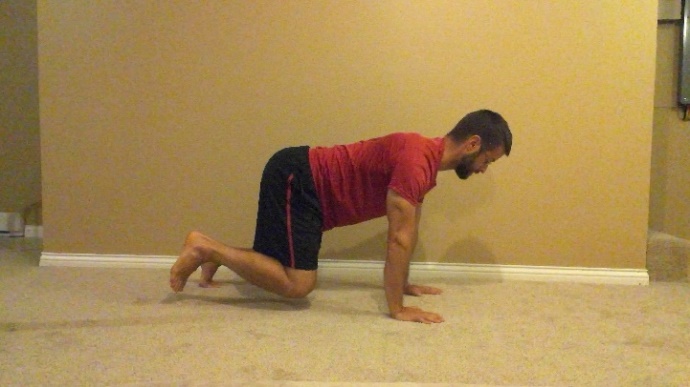


3. Static with hand and foot lift – Set Static Beast then perform a contralateral limb lift (e.g., right hand and left foot), again lifting each limb only high enough to slide a piece of paper under while not allowing any other body movements.


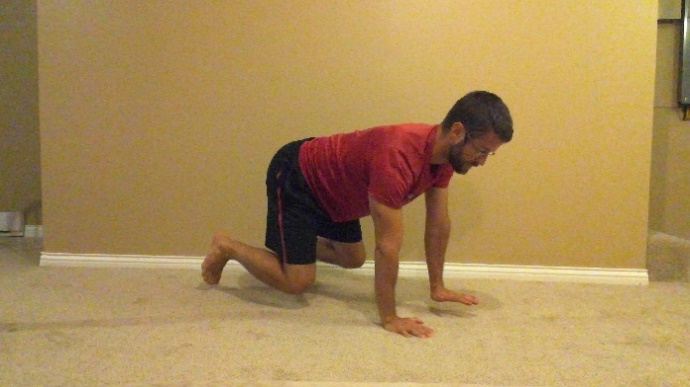


*Crab Activations*

1. Static crab – begin in a seated position with feet just outside hip width, hands shoulder width and fingers pointed back. Position hips midway between feet and hands. Then press hands into the ground, depressing shoulders and slightly elevating the hips off the floor.


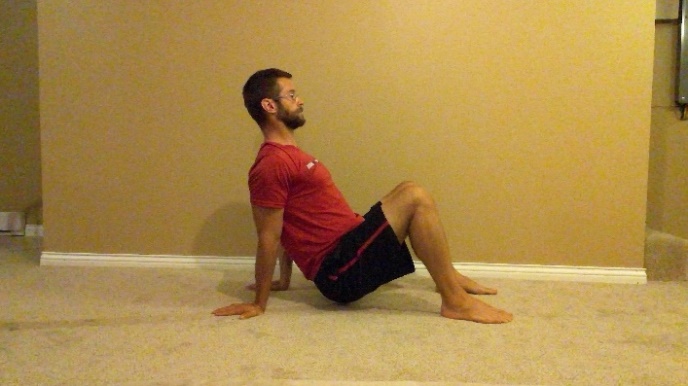


2. Static with single limb lift – Set Static Crab then lift one limb (hand or foot) off the ground without allowing any additional body movements.


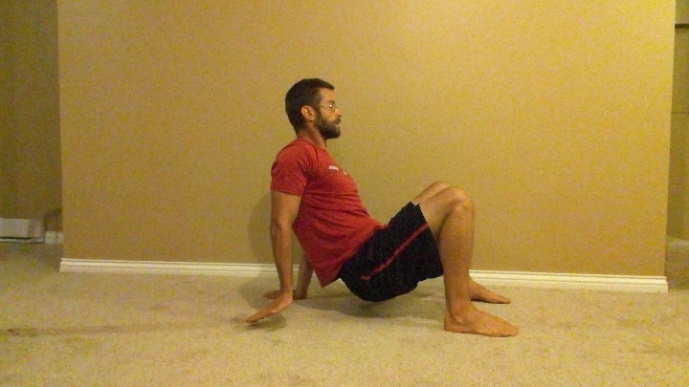


3. Static with hand and foot lift – Set Static Crab then perform a contralateral limb (e.g., right hand and left foot) lift without allowing additional body movements.


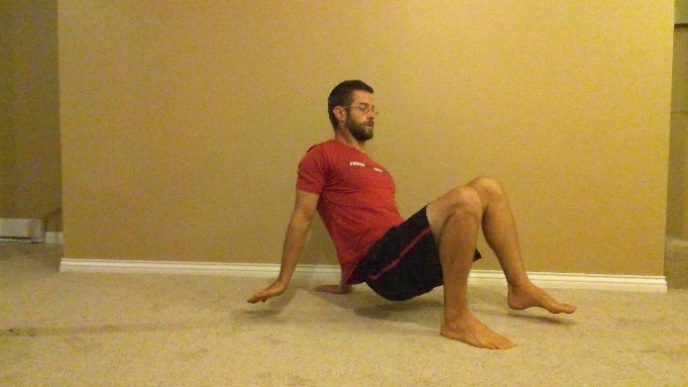


**Form Specific Stretches:** Movements in this component of AF are designed to encourage flexibility and stability throughout the kinetic chain. All movements within this component begin in one of the three base positions (Beast, Crab or Ape) and move through various end ranges of motion. These movements can be used as conditioning on their own or integrated into a flow.

*Loaded Beast* – Set Static Beast then rock hips back until back of hips are touching (or nearly touching) heels. Keep knees off the ground and the shins parallel to the floor.


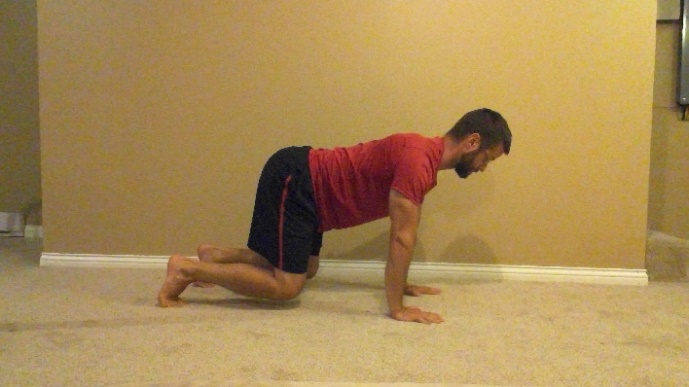

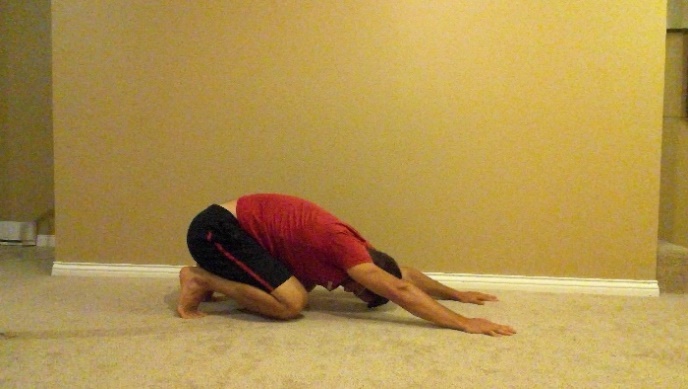


*Wave unload* – Begin in Loaded Beast. Lift the hips towards the ceiling extending the knees. Once the knees are fully extended tuck the chin and begin driving the hips and shoulders forward over the hands. As the shoulders move past the hands begin to drop the hips and move into spinal extension. Finish by lifting the chin and looking towards the ceiling. Reverse the wave and return to the Loaded Beast position.


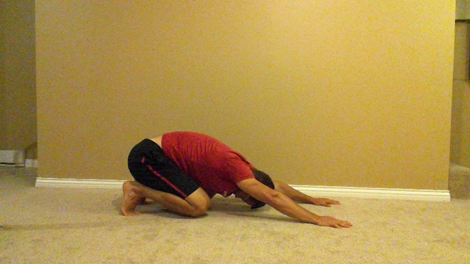

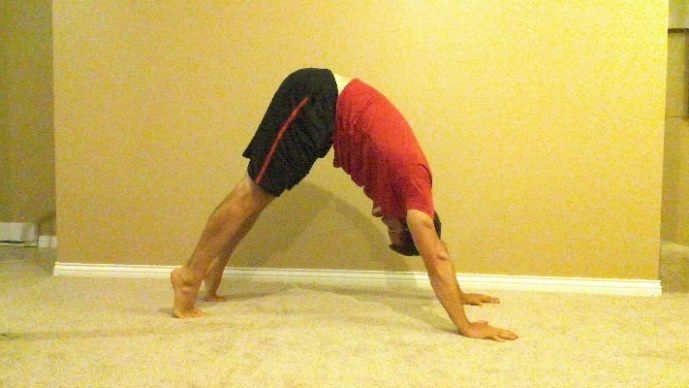


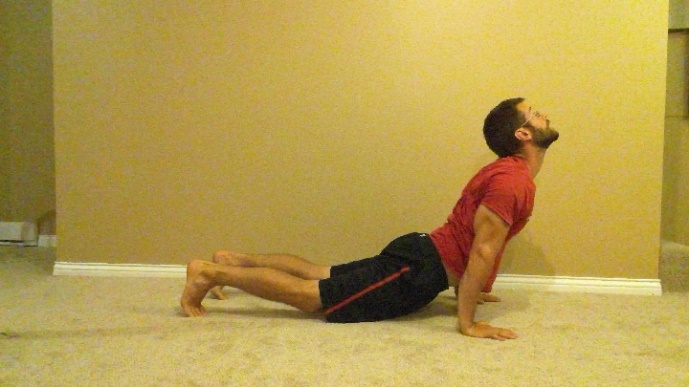


*Scorpion Reach* – Begin in Loaded Beast. Drive the hips and trunk forward, carrying the reaching leg with you (as in the Beast Reach) towards the opposite wrist and forearm. The reaching foot then circles out and up towards the ceiling. The head drops down between the arms and the reaching leg remains bent at 90 degrees. Finish by reversing the movement, bringing the reaching leg towards the opposite forearm before returning to Loaded Beast.


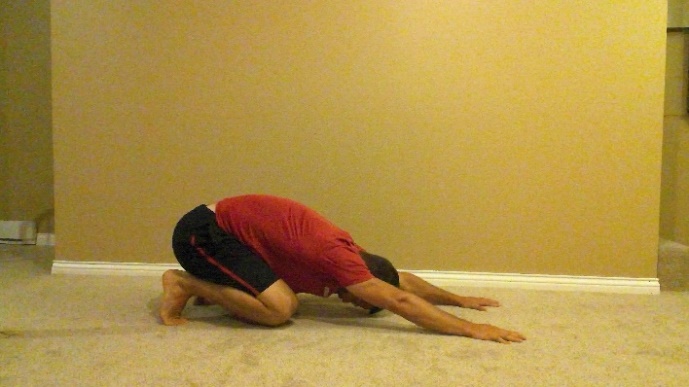

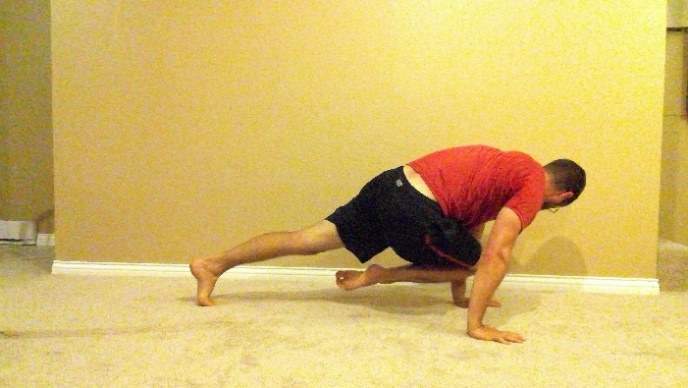


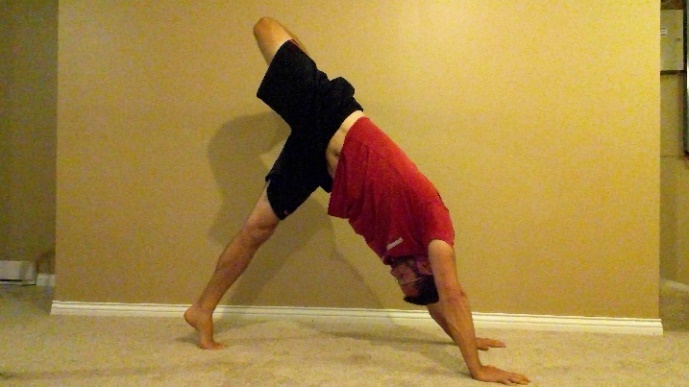


*Crab Reach* – Begin in Static Crab. Move the reaching hand in front of midline of the face. Drive through the heels pressing the hips towards the ceiling. Once the hips are in line with (or higher than) the knees and shoulder, let the reaching arm travel overhead and reach towards the ground. The eyes should follow the reaching hand.


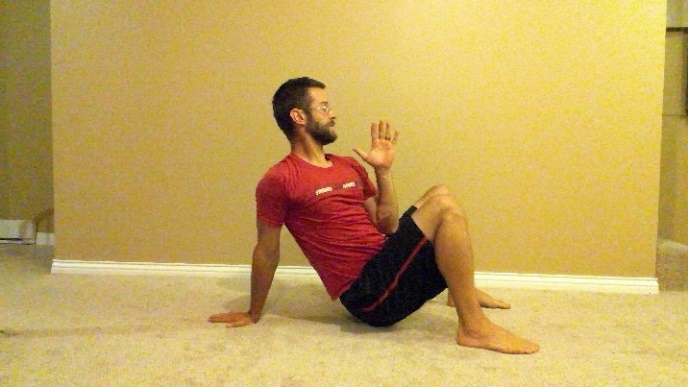

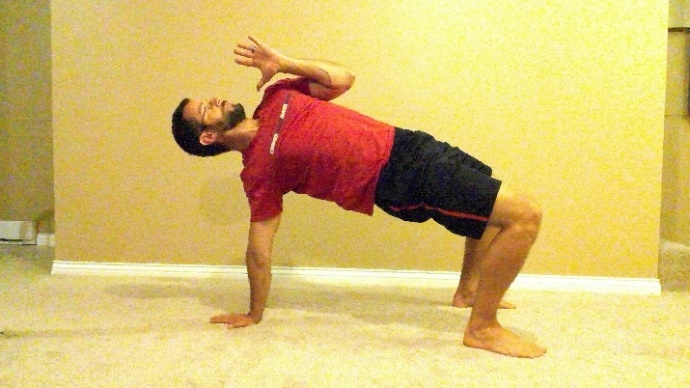


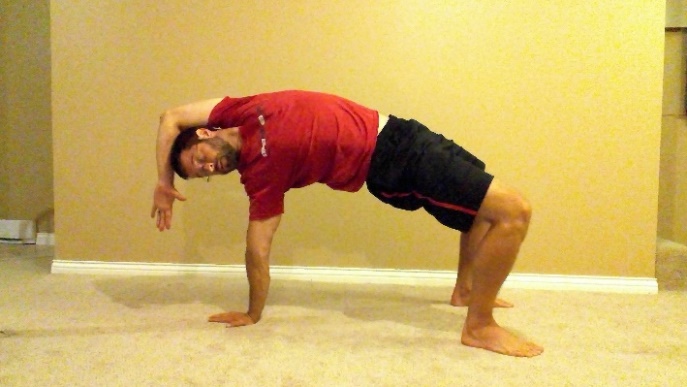


**Switches and Transitions:** Switches and transitions make up the bulk of the movements used in a flow. They are whole-body dynamic movements originated from one of the three base positions mentioned earlier and can be easily linked to other movements within this component. Movements in this component can be used as conditioning on their own or integrated into a flow.

*Underswitch* – From Crab position, lift the moving leg limb and contralateral hand. Drive through the toes of the base foot. Pull the moving leg under and in towards the midline along with the traveling arm as you rotate towards the Static Beast position. Both moving limbs should land and the same time. Underswitches can be performed from the Static Crab or Beast position.


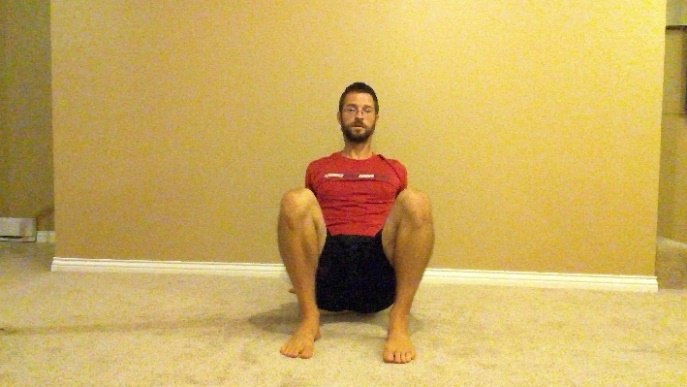

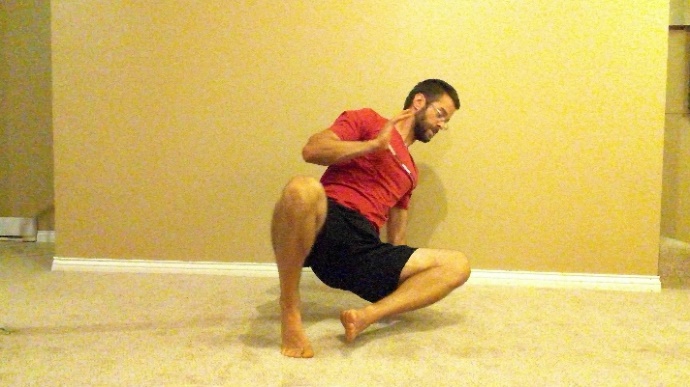


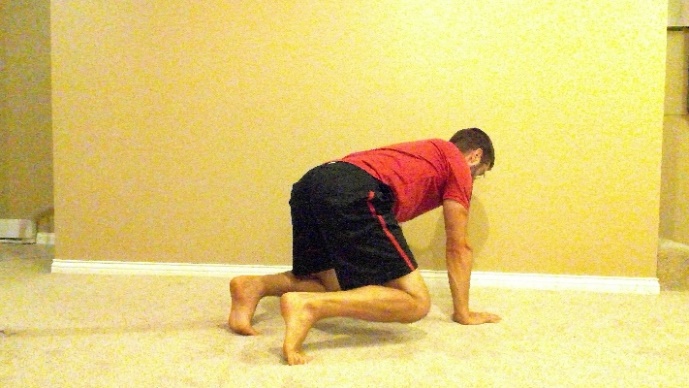


*Side Kickthrough* – Begin in Static Beast. Lift the moving foot and opposite hand off the ground to initiate the movement. Rotate toward the kicking direction. When base foot is perpendicular to base hand, drop the heel to stop rotation. Extend moving leg, point toes and externally rotate at the hip. Draw the opposite elbow back at shoulder height to finish with the back of the hand towards the face.


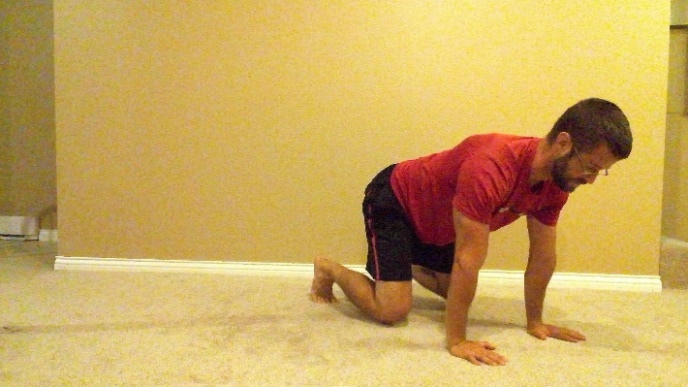

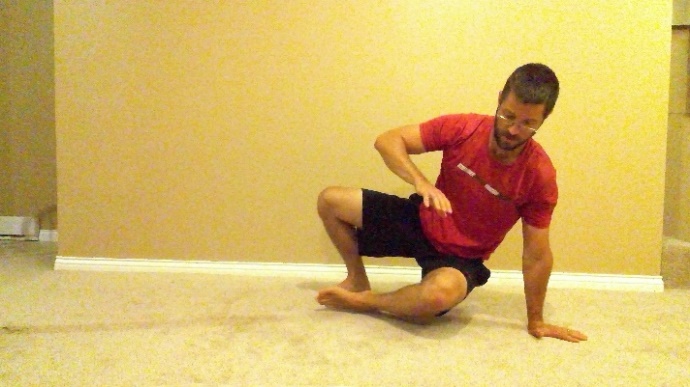


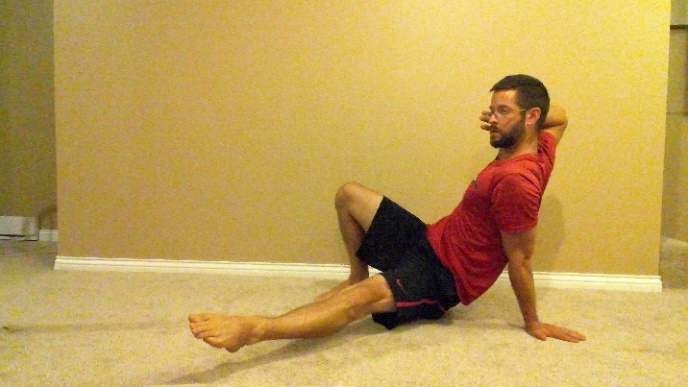


*Jumping Side Kickthrough* – Begin in a Side Kickthrough position. Drive the ball of the base foot into the ground, elevating the heel. Retract the kicking leg and bring the traveling hand towards the ground. Hop to the 3-Point Modified Beast position and then perform a Side Kickthrough to the other side.


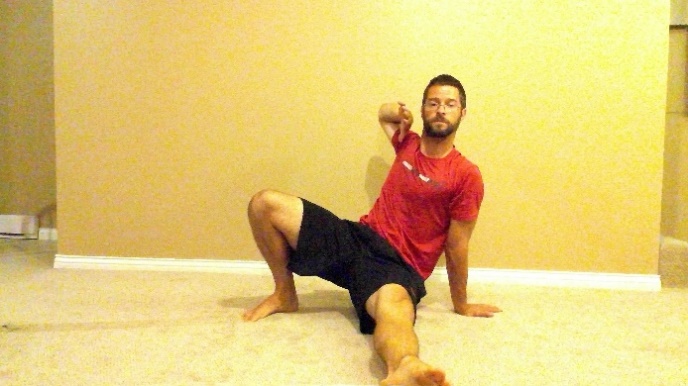

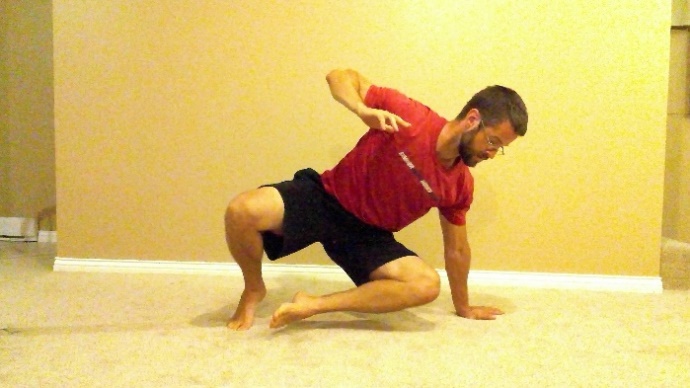


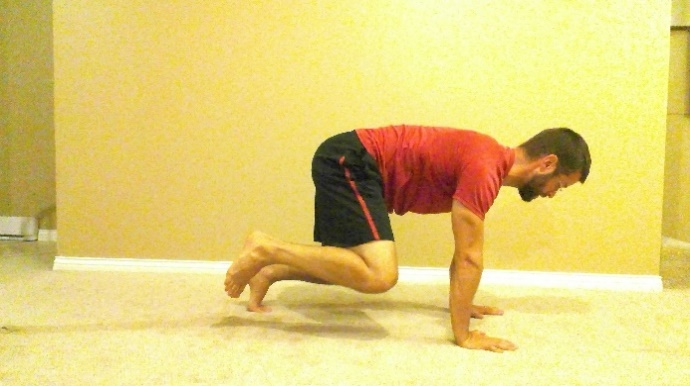

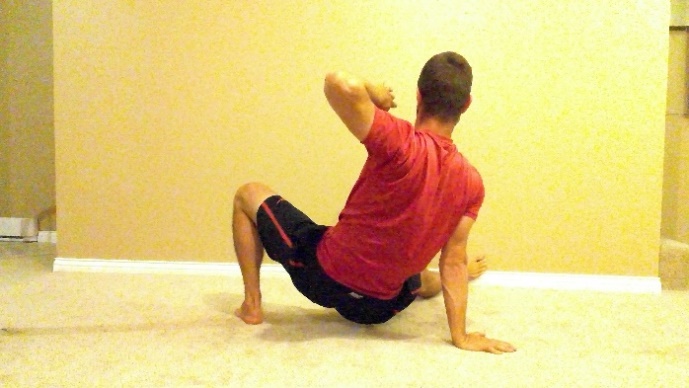


*Full Scorpion* – Begin in Static Crab. Initiate the movement by performing a normal Underswitch. The traveling arm lands but the traveling leg continues to rotate up and out keeping the knee bent at 90 degrees. As the leg travels up the head drops between the arms bringing the gaze to the base foot while the base leg begins to extend. The traveling leg then continues to rotate reaching for the ground. As the traveling leg gets closer to the ground it pulls the traveling arm off the ground. Once the travel leg hits the ground, the hips drop and the traveling arm moves into a guard position. This is known as the Modified Crab position.


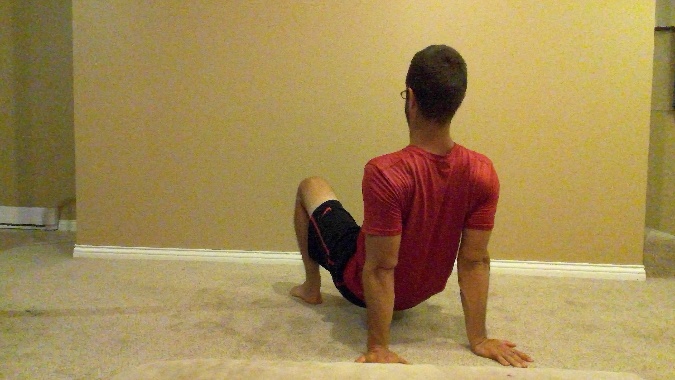

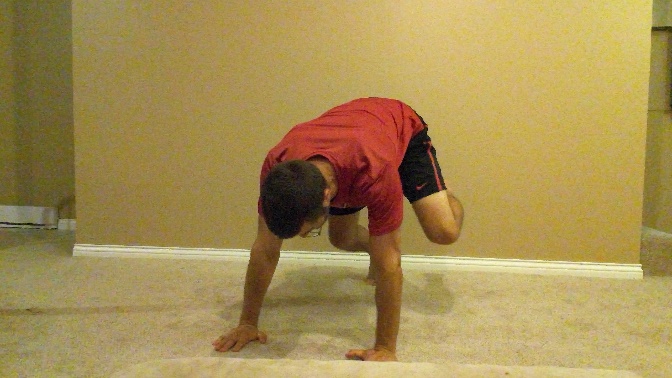


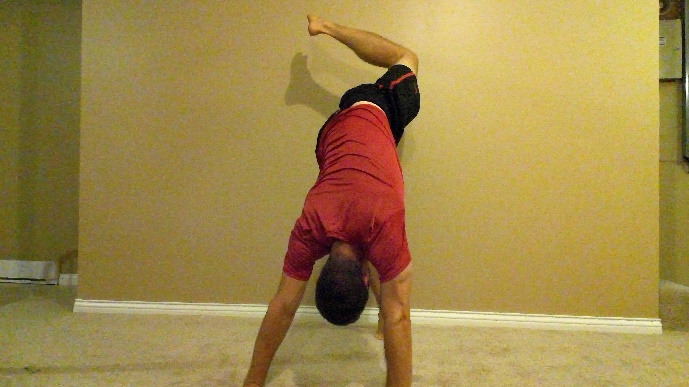

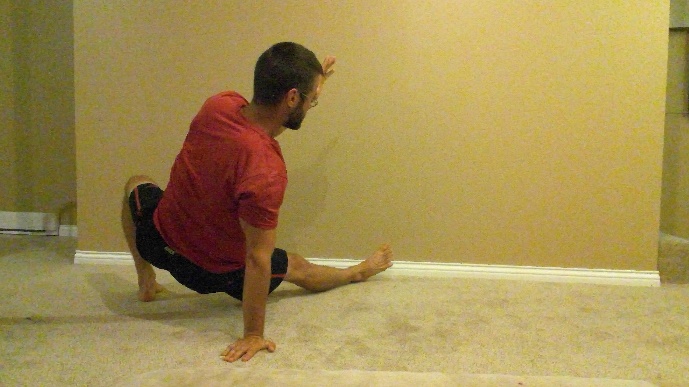


*Front Step and Front Step Through* – Begin in Loaded Beast. Drive forward toward the hands and move the traveling leg to replace the same side hand. The same side hand simultaneously lifts and pulls toward the same side shoulder. This is the Front Step. From here pull the back leg forward through the gap between the base hand and stepping foot. Kick the back leg through, extending the knees and toes and externally rotating at the hip. Simultaneously pull the opposite hand into a guard position in front of the torso and face.


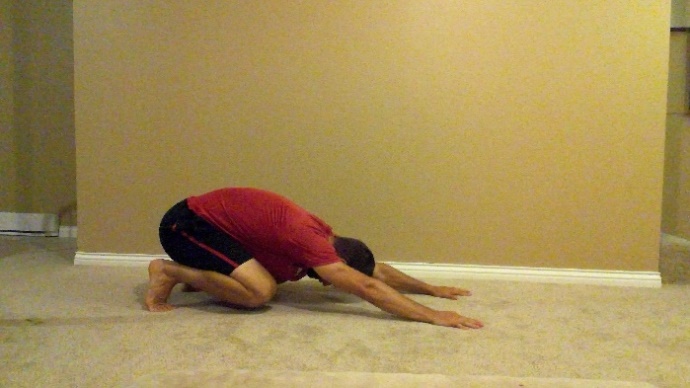

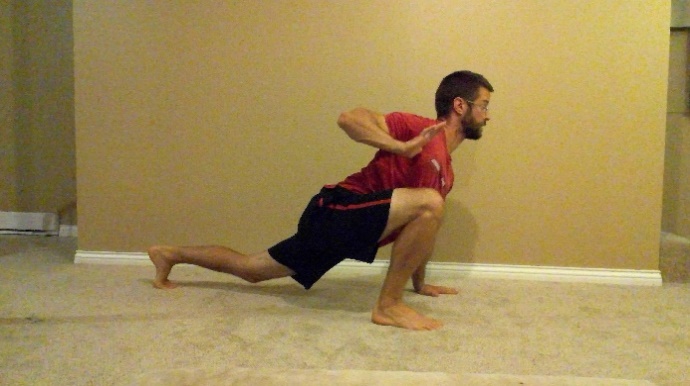


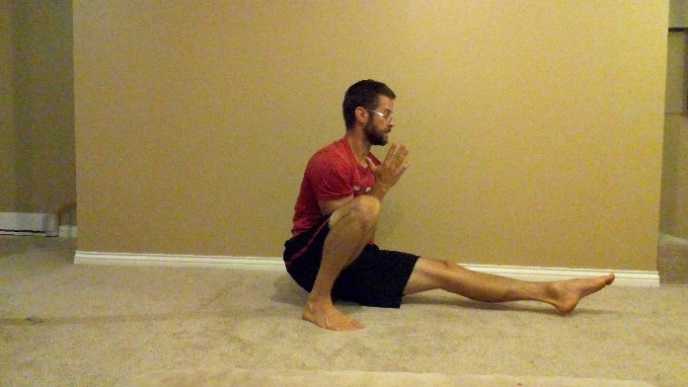


**Flow:** Flows are choreographed sequences of switches and transitions, form specific stretches and traveling forms (not used in this class) linked together to create a sequence of continuous motion. In beginner level classes, flows are designed using movements taught/practiced from earlier components and completed for several repetitions.
