# Supplementary material for: A comparison of the energy demands of quadrupedal movement training to walking
Source: Front Sports Act Living. 2022 Oct 13;4:992687. doi: 10.3389/fspor.2022.992687 (PMC9606455; doi:10.3389/fspor.2022.992687)
Supplement: Supplementary file 2 [file Data_Sheet_2.docx]

**Supplemental Digital Content (SDC)**

Supplementary Tables and Results

Table S1. Comparison of respiratory values across conditions

| **Variable** | **SPTM** | **HRTM** | **AF** | ***P*-Value** |
| --- | --- | --- | --- | --- |
| **VO_2_ (L^.^min^-1^)** | 1.1 ± 0.2 | 1.6 ± 0.5*‡ | 1.4 ± 0.4* | Interaction; *P*=0.017, η^p2^ =0.152 Session; *P*<001, η^p2^ =0.601  Gender; *P*<0.001, η^p2^ =0.605 |
| **Male** | 1.2 ± 0.1• | 1.9 ± 0.4• | 1.7 ± 0.2• |  |
| **Female** | 0.9 ± 0.2 | 1.3 ± 0.3 | 1.1 ± 0.3 |  |
| **VO_2_ (ml^.^kg^.^min^-1^)** | 14.9 ± 2.3 | 21.3 ± 3.6*‡ | 19.0 ± 3.5* | Interaction; *P*=0.081, η^p2^ =0.089 Session; *P*<001, η^p2^ =0.614  Gender; *P*<0.049, η^p2^ =0.136 |
| **Male** | 14.8 ± 2.1 | 22.7 ± 3.6 | 20.2 ± 3.4• |  |
| **Female** | 15.0 ± 2.6 | 20.1 ± 3.3 | 17.6 ± 3.3 |  |
| **VCO_2_ (L^.^min^-1^)** | 0.9 ± 0.2 | 1.3 ± 0.4* | 1.2 ± 0.3* | Interaction; *P*=0.003, η^p2^ =0.195 Session; *P*<001, η^p2^ =0.636  Gender; *P*<0.001, η^p2^ =0.612 |
| **Male** | 1.0 ± 0.1• | 1.6 ± 0.3• | 1.5 ± 0.2• |  |
| **Female** | 0.8 ± 0.2 | 1.1 ± 0.3 | 1.0 ± 0.3 |  |
| **RER** | 0.8 ± 0.1 | 0.8 ± 0.1 | 0.9 ± 0.1*† | Interaction; *P*=0.259, η^p2^ =0.049 Session; *P*<001, η^p2^ =0.439  Gender; *P*<0.167, η^p2^ =0.070 |
| **Male** | 0.8 ± 0.0 | 0.9 ± 0.0• | 0.9 ± 0.1 |  |
| **Female** | 0.8 ± 0.1 | 0.8 ± 0.0 | 0.9 ± 0.1 |  |
| **RR (br^.^min^-1^)** | 25.6 ± 3.6 | 29.6 ± 4.9* | 34.3 ± 4.1*† | Interaction; *P*=0.821 η^p2^ =0.007 Session; *P*<001, η^p2^ =0.717  Gender; *P*<0.423, η^p2^ =0.024 |
| **Male** | 24.8 ± 3.8 | 28.9 ± 5.5 | 34.0 ± 3.1 |  |
| **Female** | 26.1 ±3.5 | 30.3 ± 4.3 | 34.5 ± 5.0 |  |
| **Ve (L^.^min^-1^)** | 26.7 ± 5.0 | 39.9 ± 10.2* | 41.8 ± 8.7* | Interaction; *P*=0.008, η^p2^ =0.194 Session; *P*<001, η^p2^ =0.808  Gender; *P*<0.001, η^p2^ =0.612 |
| **Male** | 30.5 ± 4.0• | 46.8 ± 9.3• | 49.1 ± 4.6• |  |
| **Female** | 23.5 ± 3.2 | 33.5 ± 6.0 | 35.0 ± 5.8 |  |
| **Tv (L)** | 1.1 ± 0.3 | 1.4 ± 0.5*‡ | 1.2 ± 0.3* | Interaction; *P*=0.071, η^p2^ =0.093 Session; *P*<001, η^p2^ =0.407  Gender; *P*<0.001, η^p2^ =0.515 |
| **Male** | 1.3 ± 0.3• | 1.7 ± 0.5• | 1.4 ± 0.2• |  |
| **Female** | 0.9 ± 0.2 | 1.1 ± 0.2 | 1.0 ± 0.2 |  |

Data are Mean ± SD. *Note*. SPTM = self-paced treadmill walking; HRTM = heart-rate matched treadmill walking; AF = Animal Flow; HR = heart rate; RER = respiratory exchange ratio; RR = respiratory rate; TV = tidal volume; RPE = rating of perceived exertion (OMNI rating of exertion); Effect sizes: 0.01 = small; 0.06 = moderate; ≥ 0.14 = large. *= significantly greater than SPTM; †= significantly greater than HRTM; ‡= significantly greater than AF; •= significantly greater than females; °=significantly greater than males

Table S2. Comparison of respiratory values across AF segments

| Variable | WM | ACT | FS | STs | FLOW | *P*-value |
| --- | --- | --- | --- | --- | --- | --- |
| VO_2_ (L^.^min^-1^) | 0.6 ± 0.2 | 1.0 ± 0.2* | 1.3 ± 0.4*† | 1.5 ± 0.4*†‡ | 1.8 ± 0.8*†‡◊ | Interaction; *P*<0.001, η^p2^ =0.305  Session; *P*<001, η^p2^ =0.895  Gender; *P*<0.001, η^p2^ =0.609 |
| Males | 0.7 ± 0.2▪ | 1.2 ± 0.1▪ | 1.6 ± 0.2▪ | 1.9 ± 0.2▪ | 2.2 ± 0.4▪ |  |
| Females | 0.5 ± 0.0 | 0.8 ± 0.2 | 1.0 ± 0.3 | 1.2 ± 0.3 | 1.4 ± 0.4 |  |
| VO_2_ (ml^.^kg^.^min^-1^) | 8.7 ± 2.1 | 14.1 ± 2.1* | 18.0 ± 3.4*† | 21.5 ± 4.1 *†‡ | 24.6 ± 5.8*†‡◊ | Interaction; *P*=0.097, η^p2^ =0.090  Session; *P*<001, η^p2^ =0.898  Gender; *P*=0.012, η^p2^ =0.213 |
| Males | 9.6 ± 2.5▪ | 15.0 ± 1.9▪ | 19.4 ± 3.0▪ | 23.4 ± 3.5▪ | 27.1 ± 5.3▪ |  |
| Females | 7.9 ± 1.2 | 13.3 ± 2.0 | 16.7 ± 3.2 | 19.7 ± 3.9 | 22.3 ± 5.5 |  |
| VCO_2_ (L^.^min^-1^) | 0.5 ± 0.2 | 1.0 ± 0.2* | 1.1 ± 0.3*† | 1.4 ± 0.4*†‡ | 1.6 ± 0.5*†‡◊ | Interaction; *P*=0.004, η^p2^ =0.214  Session; *P*<001, η^p2^ =0.863  Gender; *P*<0.001, η^p2^ =0.596 |
| Males | 0.7 ± 0.2▪ | 1.2 ± 0.1▪ | 1.4 ± 0.2▪ | 1.7 ± 0.3▪ | 1.9 ± 0.4▪ |  |
| Females | 0.4 ± 0.0 | 0.8 ± 0.2 | 0.9 ± 0.2 | 1.1 ± 0.3 | 1.3 ± 0.4 |  |
| RER | 0.8 ± 0.1 | 0.9 ± 0.1*‡◊ | 0.9 ± 0.1* | 0.9 ± 0.1* | 0.9 ± 0.1* | Interaction; *P*=0.500, η^p2^ =0.026  Session; *P*<001, η^p2^ =0.406  Gender; *P*=0.959 η^p2^ =0.001 |
| Males | 0.9 ± 0.1 | 0.9 ± 0.1 | 0.9 ± 0.1 | 0.9 ± 0.1 | 0.9 ± 0.1 |  |
| Females | 0.8 ± 0.1 | 1.0 ± 0.1 | 0.9 ± 0.1 | 0.9 ± 0.1 | 0.9 ± 0.1 |  |
| V_E_ (L^.^min^-1^) | 17.0 ± 4.8 | 30.2 ± 6.6* | 38.6 ± 8.9*† | 45.8 ± 10.1*†‡ | 57.8 ± 13.0*†‡◊ | Interaction; *P*<0.001, η^p2^ =0.331  Session; *P*<001, η^p2^ =0.948  Gender; *P*<0.001, η^p2^ =0.601 |
| Males | 20.4 ± 4.5▪ | 34.7 ± 4.8▪ | 45.0 ± 5.7▪ | 53.8 ± 5.8▪ | 67.8 ± 9.7▪ |  |
| Females | 13.9 ± 2.4 | 26.0 ± 5.2 | 32.6 ± 6.9 | 38.3 ± 6.9 | 48.5 ± 7.8 |  |
| RR (b^.^min^-1^) | 20.1 ± 2.6 | 27.9 ± 3.9* | 34.2 ± 4.5*† | 36.2 ± 4.8*†‡ | 43.6 ± 5.6*†‡◊ | Interaction; *P*=0.977, η^p2^ =0.002  Session; *P*<001, η^p2^ =0.926  Gender; *P*=0.910 η^p2^ =0.001 |
| Males | 19.9 ± 2.4 | 27.7 ± 3.2 | 34.1 ± 3.6 | 36.2 ± 3.4 | 43.7 ± 4.8 |  |
| Females | 20.2 ± 2.8 | 27.9 ± 4.5 | 34.4 ± 5.3 | 36.3 ± 4.8 | 43.5 ± 6.4 |  |
| TV (L) | 0.9 ± 0.2 | 1.1 ± 0.2* | 1.1 ± 0.3* | 1.3 ± 0.3*†‡ | 1.3 ± 0.3*†‡◊ | Interaction; *P*=0.097, η^p2^ =0.080  Session; *P*<001, η^p2^ =0.798  Gender; *P*<0.001 η^p2^ =0.551 |
| Males | 1.1 ± 0.2▪ | 1.3 ± 0.2▪ | 1.3 ± 0.2▪ | 1.5 ± 0.2▪ | 1.6 ± 0.2▪ |  |
| Females | 0.7 ± 0.1 | 0.9 ± 0.2 | 1.0 ± 0.2 | 1.1 ± 0.3 | 1.1 ± 0.2 |  |

Data are Mean ± SD. *Note*. AF = Animal Flow; WM = wrist mobilizations; ACT = activations; FS = form stretches; STs = switches and transitions; FLOW = choreographed flow; RER = respiratory exchange ratio; RR = respiratory rate; TV = tidal volume; RPE = rating of perceived exertion (OMNI rating of exertion); Effect sizes: 0.01 = small; 0.06 = moderate; ≥ 0.14 = large. *= significantly greater than WM; †= significantly greater than ACT; ‡= significantly greater than FS; ◊= significantly greater than STs; •= significantly greater than FLOW; ▪= significantly greater than females; °=significantly greater than males
